# Patient functionality and walking speed after discharge from the intensive care unit

**DOI:** 10.5935/0103-507X.20190066

**Published:** 2019

**Authors:** Priscila Becker da Silva, Laura Jurema dos Santos

**Affiliations:** 1 Residência Multiprofissional em Saúde do Adulto e Idoso, Universidade Luterana do Brasil - Canoas (RS), Brasil.; 2 Curso de Fisioterapia, Universidade Luterana do Brasil - Canoas (RS), Brasil.

**Keywords:** Critical care, Walk test, Early ambulation, Muscle strength, Exercise, Patient care, Patient discharge, Intensive care units

## Abstract

**Objective:**

To measure and compare the functionality of patients after discharge from the intensive care unit and at the time of hospital discharge.

**Methods:**

Quantitative study of a prospective cohort performed between August of 2016 and December of 2017 at a university hospital. A 10-meter walk test was performed at 2 timepoints: after discharge from the intensive care unit and prior to hospital discharge. The data were analyzed using Student's t-test and Pearson or Spearman correlation. Statistical Package for Social Science (SPSS) version 21.0 was used for the analysis, and p ≤ 0.05 was adopted as the level of significance.

**Results:**

Forty patients, with a mean age of 57.1 ± 12.2 years and with a predominance of males (60%), were evaluated. For the post-intensive care unit test, a mean speed of 0.48m/s was observed, and for the pre-hospital discharge test, there was an increase to 0.71m/s, evidencing functional evolution during the hospital stay (p < 0.001).

**Conclusion:**

There was significant improvement in walking speed at the time of hospital discharge when compared to the walking speed at the time of intensive care unit discharge.

## INTRODUCTION

Patients with intensive care unit (ICU)-acquired muscle weakness have difficulty not only returning to spontaneous ventilation but also moving in general.^(1)^ Intensive care unit stays promote a marked decline in the quality of life of individuals.^(2)^ Studies indicate that recovery of quality of life, physical capacity, general health and social health of patients is incomplete, even 6 months after hospital discharge.^(2)^ Possible impairments include muscle weakness, cognitive impairment, psychological difficulties, reduced physical function, such as in activities of daily living (ADLs), and decreased quality of life.^(3)^ Early interventions, such as mobilization or active exercise, or both, can reduce the impact of critical illness sequelae.^(3)^ Such deleterious effects in critically ill patients are consequences of prolonged hospital stays and of periods of mechanical ventilation (MV).^(4)^

A study by França et al.^(5)^ showed that the recommendations for orthostasis and ambulation in patients under MV increase the functional status and reduce neuromuscular complications, boosting the management of critically ill patients. This need was evidenced by Curzel et al.,^(6)^ who found that after discharge from the ICU, the lowest score on the Functional Independence Measure (FIM) was in the locomotion criterion, restricting functional independence.

The walk test, such as the 10m walk test, is a relatively simple and reproducible method that allows assessing the functional capacity of patients.^(7)^ The walk test can be used to estimate functionality, chronic disease, general health and skeletal muscle mass, depending on the variation in the rhythm dictated by the patient profile, given that age, sex and ethnicity affect the speed obtained.^(8,9)^

Fritz et al.,^(10)^ exploring the correlation between the effects of prolonged ICU stay and functionality tests, conducted a study using the 10m walk test; the conclusion was that a walking speed below 0.6m/s is associated with lower functionality, a higher risk of hospital readmission, and a greater need for the use of rehabilitation services and walking aids and that walking speeds above 1m/s are associated with functional independence, a lower risk of falling and walking without assistance, which may be one of the factors considered for hospital discharge. Another study, conducted with 34,485 elderly people from different countries, concluded that life expectancy increases exponentially according to the speed achieved during walking - approximately 0.8m/s.^(11)^ Notably, there are tests similar to the 10-m walk test that use alternative distances, such as the 6-m walk test, which was used to measure walking speed of 110 hospitalized elderly patients; those with walking speeds above 1m/s had a lower correlation with risk of falling and greater functionality.^(12)^

Given the available literature on the subject and other references similar to the subject of interest, the aim of the present study was to measure and compare the functionality of patients after discharge from the ICU and prior to hospital discharge and to correlate walking speed with length of hospital stay and ICU stay and with the duration of MV.

## METHODS

This was a quantitative prospective cohort study conducted from August 2016 to December 2017 at *Hospital Universitário de Canoas*. The sample size was calculated based on the results of Oliveira et al.^(13)^ Considering a significance level of 5%, 90% power and a minimum correlation coefficient of 0.6 between length of stay and walking speed, the sample size estimated by the statistical software Programs for Epidemiologists for Windows (WinPEPI), version 11.43, was a minimum total of 25 patients, but it was decided to evaluate 40 patients, considering the risk of loss to follow-up, in order to maintain a safety margin in the total sample.

Patients older than 18 years, of both sexes and hospitalized in the ICU for more than 48 hours were evaluated. After explaining the study, all participants signed an informed consent form, drafted according to the Guidelines and Norms Regulating Research With Human Beings, established in the Brazilian National Health Council Resolution 466/12. In cases in which the patient was unable to sign the form, the relative responsible was asked to do so. Patients with hemodynamic instability, neurological impairment, orthopedic trauma and cognitive deficits were excluded. The participants received the physical therapy protocol of the hospital, and the study did not interfere in the performance of the institution's service.

Possible participants were selected by analyzing the medical records of patients admitted to *Hospital Universitário de Canoas*. Subsequently, when invited to participate in the study, the patients were informed about its nature and procedures, such as how the tests would be conducted, the risks and the benefits. Next, personal data were collected. The first application of the 10m walk test occurred in the first 24 hours after discharge from the ICU, and the second was performed 24 hours prior to hospital discharge. The test was performed in a hallway, at least 20m in length, with little circulation of people. Three chairs were placed along the walking route to be used only if the patient needed to rest during the walk (one at the beginning, one in the middle and one at the end of the distance to be covered). All tests were conducted by the same researcher.

The first 5m of the course allowed for gait acceleration and was not considered in the speed achieved by the patient. The next 10m was the distance relevant for the study. The final 5m allowed for gait deceleration,^(8)^ and speed was not recorded. The 3 distances were marked on the floor with the help of a tape measure. Each participant performed the test 3 times at each timepoint, and the mean of the 3 values was used.^(7)^ Before each repetition of the route, vital signs of each patient were recorded.

Walking speed, in meters/second, was recorded using a stopwatch while the patient traversed the 10 central meters. At the end of the 3 attempts, the vital signs of each patient were again recorded. All study participants continued receiving routine physiotherapeutic care, i.e., 2 daily visits.

### Data analysis

The quantitative variables are expressed as the mean and standard deviation; the median is included in cases of asymmetry. Qualitative variables are expressed as absolute and relative frequencies. Student's t-test for paired samples was used to compare means between timepoints. To evaluate the correlations between length of hospital stay, length of ICU stay and duration of MV with 10m walking speed, the Pearson or Spearman linear correlation tests were used. The significance level adopted was 5% (p < 0.05), and the analyses were performed in the Statistical Package for the Social Sciences (SPSS), version 21.0.

This study was approved by the Ethics and Research Committee of *Universidade Luterana do Brasil* (opinion n. 1620.638).

## RESULTS

**Figure 1:**
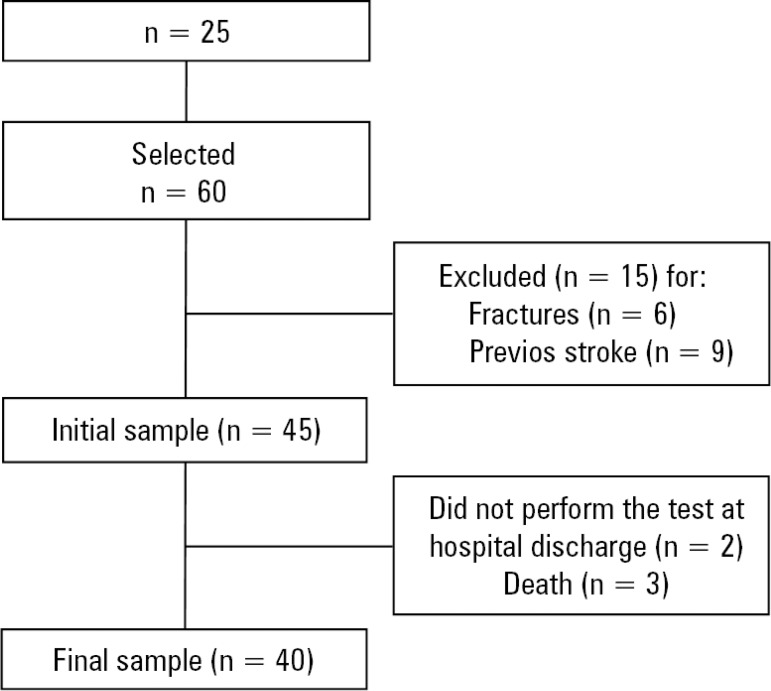
Flowchart representing the individuals who participated in the study and those lost to follow-up.

Between August 2016 and December 2017, 60 patients admitted to *Hospital Universitário de Canoas* were consecutively followed-up; 15 met the exclusion criteria, such as previous neurological deficit or recent fracture, 3 patients died, and 2 did not perform the test prior to hospital discharge because they were discharged without prior scheduling, causing them to not be followed-up at the second collection time. Thus, 40 patients were included in the final sample, which consisted of clinical, urgent care and elective surgery patients (Figure 1).

Table 1 shows the characteristics of the sample, whose mean age was 57.1 ± 12.2 years, with a predominance of males (60%).

**Table 1:** Sample characteristics

Variable	
Age (years)	57.1 ± 12.2
Sex	
Female	16 (40.0)
Male	24 (60.0)
MRS	11 (27.5)
AMI	8 (20.0)
Unstable angina	6 (15.0)
Other pathologies	15 (37.5)
Length of hospital stay (days)	16 (11 - 22)
Length of stay in the ICU (days)	4.0 ± 1.8
MV duration (days)	1 (0 - 1)

MRS - myocardial revascularization surgery; AMI - acute myocardial infarction; ICU - intensive care unit; MV - mechanical ventilation. The results are expressed as the mean ± standard deviation, n (%) or median (P25 - P75).

**Figure 2:**
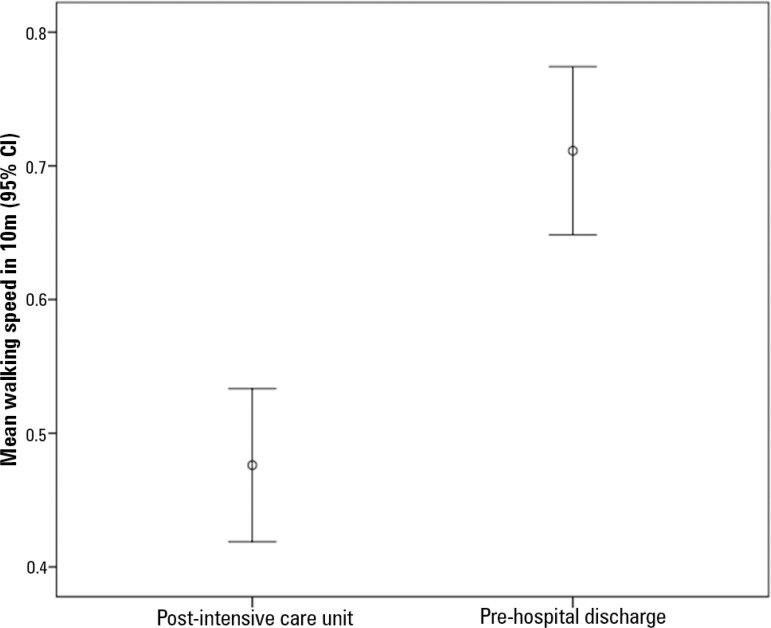
Speed on the 10-meter walk test. 95%CI- 95% confidence interval; ICU - intensive care unit. Mean ± standard deviation. Post-intensive care unit = 0.48 ± 0.18m/s; Pre-hospital discharge = 0.71 ± 0.20m/s; difference (95% confidence interval) = 0.24 (0.20 - 0.27); p < 0.001.

Figure 2 shows the comparison between the mean walking speed after ICU discharge and before hospital discharge. In the post-ICU test, the mean speed was 0.48 ± 0.18m/s, and at hospital discharge, there was an increase to 0.71 ± 0.20m/s, indicating a mean difference of 0.24m/s between the 2 times and evidencing significant functional progress during the hospital stay (p < 0.001), with a 47.9% increase in walking speed.

Table 2 shows the correlation between length of hospital stay, length of ICU stay only and duration of MV with 10m walking speed achieved by the patient, with a significant (p < 0.001) and moderate correlation between walking speed and length of hospital stay. The shorter the length of hospital stay, the faster was the walking speed achieved at the time of hospital discharge, noting that faster walking speeds were proportionally related to the length of hospital stay, given that individuals with better walking performance had a lower total length of hospital stay. There was no significant correlation between the 10m walking speed and the duration of MV, but it is suggested that the shorter the duration of MV, the faster was the walking speed achieved.

**Table 2:** Correlation between the 10m walk test speed and the length of hospital stay, intensive care unit stay and duration of mechanical ventilation

Variable	Walk test time		Difference
	Post-ICU	Pre-discharge	
Length of hospital stay (days)	rs = -0.625; p < 0.001	rs = -0.272; p = 0.090	rs = 0.478; p = 0.002
Length of ICU stay (days)	r = 0.060; p = 0.715	r = 0.175; p = 0.279	r = 0.212; p = 0.190
MV duration (days)	rs = -0.374; p = 0.017	rs = -0.203; p = 0.209	rs = 0.166; p = 0.306

ICU - intensive care unit; MV - mechanical ventilation.

## DISCUSSION

The present study showed the progression of functionality of patients admitted to *Hospital Universitário de Canoas*, with a higher walking speed at the time of hospital discharge than at the time of discharge from the ICU.

The sample characteristics were consistent with the study by Cordeiro et al.^(14)^ with 64 patients, who were predominantly male (51.5%) with mean age of 57.2 ± 14.06 years.

The mean length of hospital stay was similar to that reported by Yassin et al.,^(15)^ 11.8 days of hospitalization for patients diagnosed with community-acquired pneumonia, which differed from the length of hospital stay found in the study by Suarez-de-la-Rica et al.,^(16)^ according to which patients with acute renal failure had a mean length of hospital stay of 26.9 days.

Chen et al.^(17)^ conducted a study with patients with acute respiratory distress syndrome and A type aortic dissection receiving tidal volume ventilation (6 - 8mL/kg) and found a 17-day difference in length of stay in the ICU when compared with a control group. This time differed from that of patients who stayed in the ICU for a mean of 22 hours after transfemoral transcatheter aortic valve replacement described by Babaliaros et al.,^(18)^ which corroborates the time found in the present study. A study by Klompas et al.^(19)^ found a mean MV duration of 6 days in patients undergoing anesthesia, which was quite different from that found by Li et al.,^(20)^ whose critically ill patients had a mean MV duration of 51 days.

The speed on the 10m walk test, according to the execution proposed by Fritz et al.,^(10)^ is matched with other walk tests, such as the 6 minute walk test (6MWT) and the timed up and go (TUG) test.^(21)^ Starting from this premise, a study with a population of 77 individuals with a mean age of 59 years showed a walking speed of 0.75m/s in the 10m walk test.^(22)^ A multicenter study by Huisman et al.^(23)^ conducted with 280 patients with a mean age of 70 years and admitted to the ICU concluded that a time longer than 20 seconds to perform the TUG implies greater risk during hospitalization. The TUG test was used in a study conducted with 68 hospitalized elderly patients with a mean age of 70.4 ± 7.7 years, and the mean completion time was ≥ 10.85 seconds; greater time spent on the test was correlated with individuals with greater cognitive and functional decline. There were no significant correlations between the TUG test time and the mean length of hospital stay, as in the present study.^(24)^ This finding differs from that reported by Cordeiro et al.,^(14)^ who found a correlation between length of hospital stay and walking speed (r = 0.27; p = 0.02) in their study with patients undergoing cardiac surgery; however, they found no significant correlation with MV duration.

Comparing the 2 means obtained at the different times in the present study, the walking speed of 0.71m/s is close to that found by Studenski et al.,^(11)^ and a speed greater than 0.8m/s is within the expected parameters for the average life expectancy for the population over 65 years. However, given that the mean age of the sample in question was 57.1 years, this result indicates impaired functionality.

In a study with 64 hemiparetic patients, walking speed was a powerful indicator of function and prognosis after stroke. A speed < 0.4m/s is observed for household ambulators, 0.4 to 0.8m/s is observed for patients with limited community ambulation, and > 0.8m/s is observed for patients with independent community ambulation, quantifying the functionality of patients after stroke.^(25)^ Ursin et al.,^(26)^ in a study with 180 poststroke patients, found a mean walking speed of 0.88m/s on the 10m walk test.

A study performed in Japan with 830 elderly patients applying a kinesiotherapy protocol with 1 year follow-up, comparing a control group and an intervention group, concluded that there was an increase from 0.5 to 0.6m/s in the walking speed in the intervention group, whereas in the control group, there was a decrease of 1.4m/s.^(27)^ Barbat-Artigas et al.,^(28)^ by means of muscle reinforcement, also optimized gait speed in elderly women, concluding that greater the functionality resulted in a higher gait speed.

Impaired lung function, malnutrition and muscle weakness were described as important factors for determining the physical performance of hospitalized patients, and increased respiratory rate, decreased tidal volume and hypoxemia are also limiting factors of physical capacity.^(29)^ A study comparing a group of adult individuals with patients after ICU stays between 4 and 7 days, in which the control group performed daily training walking on the ground and on a treadmill while the intervention group initially walked 5m per day and added another 5m every day, concluded that the mean walking speed of the patients was 0.78m/s, while the control group achieved 0.92m/s.^(30)^ These results contribute to the confirmation of the hypothesis by Zorowitz,^(31)^ who, after a literature review, stated that patients who do not receive adequate treatment after hospital discharge may have remnants of ICU-acquired muscle weakness for years, denoting the functional decline in this patient profile and the need for early intervention.

The majority of individuals who participated in this study (27.5%) presented with unstable angina as a reason for hospitalization, and all of them underwent myocardial revascularization surgery. According to Monteleone et al.,^(32)^ even individuals capable of walking prior to admission exhibit functional limitations in the initial postoperative period of cardiothoracic surgery, represented by a reduced walking capacity.

A limitation of this study is the small number of patients who did not meet the exclusion criteria; a large number had previous neurological injury, with gait changes, resulting in a small sample size. Another limiting factor was the fact that, given the need for application of the 10m walk test at 2 different timepoints, some patients were discharged without the test being scheduled in the electronic medical record, making it impossible to perform the measurement at hospital discharge or resulting in the nonfollow-up of these patients after hospital discharge. Another limiting factor was the lack of sufficient data to better describe the control of confounders for the association between the 10m walk test and other outcomes.

## CONCLUSION

There was significant improvement in walking speed at the time of hospital discharge compared to after the intensive care unit stay. The 10m walk test may be a functional indicator that is easily applicable in the hospital setting.
